# Evaluation of Three Morphologically Distinct Virus-Like Particles as Nanocarriers for Convection-Enhanced Drug Delivery to Glioblastoma

**DOI:** 10.3390/nano8121007

**Published:** 2018-12-05

**Authors:** Joel A. Finbloom, Ioana L. Aanei, Jenna M. Bernard, Sarah H. Klass, Susanna K. Elledge, Kenneth Han, Tomoko Ozawa, Theodore P. Nicolaides, Mitchel S. Berger, Matthew B. Francis

**Affiliations:** 1 Department of Chemistry, University of California, Berkeley, CA 94720, USA; jaf@berkeley.edu (J.A.F.); aanei.ioana@gmail.com (I.L.A.); bernarje@gmail.com (J.M.B.); sklass@berkeley.edu (S.H.K.); susannaelle@berkeley.edu (S.K.E.); kennethhan@berkeley.edu (K.H.); 2 Department of Neurological Surgery, University of California, San Francisco, CA 94158, USA; tomoko.ozawa@ucsf.edu (T.O.); Mitchel.Berger@ucsf.edu (M.S.B.); 3 Department of Pediatrics, NYU Langone Medical Center, New York, NY 10016, USA; Theodore.nicolaides@nyumc.org; 4 Materials Sciences Division, Lawrence Berkeley National Laboratories, Berkeley, CA 94720, USA

**Keywords:** virus-like particles, glioblastoma, convection-enhanced delivery, tobacco mosaic virus, bioconjugation, doxorubicin, drug delivery, protein-based nanomaterials, viral capsid

## Abstract

Glioblastoma is a particularly challenging cancer, as there are currently limited options for treatment. New delivery routes are being explored, including direct intratumoral injection via convection-enhanced delivery (CED). While promising, convection-enhanced delivery of traditional chemotherapeutics such as doxorubicin (DOX) has seen limited success. Several studies have demonstrated that attaching a drug to polymeric nanoscale materials can improve drug delivery efficacy via CED. We therefore set out to evaluate a panel of morphologically distinct protein nanoparticles for their potential as CED drug delivery vehicles for glioblastoma treatment. The panel consisted of three different virus-like particles (VLPs), MS2 spheres, tobacco mosaic virus (TMV) disks and nanophage filamentous rods modified with DOX. While all three VLPs displayed adequate drug delivery and cell uptake in vitro, increased survival rates were only observed for glioma-bearing mice that were treated via CED with TMV disks and MS2 spheres conjugated to doxorubicin, with TMV-treated mice showing the best response. Importantly, these improved survival rates were observed after only a single VLP–DOX CED injection several orders of magnitude smaller than traditional IV doses. Overall, this study underscores the potential of nanoscale chemotherapeutic CED using virus-like particles and illustrates the need for further studies into how the overall morphology of VLPs influences their drug delivery properties.

## 1. Introduction

Protein-based nanomaterials are a promising class of nanocarriers for drug delivery and diagnostic applications. These materials are formed through the self-assembly of protein monomers into larger nanoscale scaffolds of varying morphologies and properties [1,2,3,4]. Protein-based nanomaterials and specifically virus-like particles (VLPs) based on naturally occurring viruses, have demonstrated effectiveness in drug delivery and imaging applications [1,2,3,4,5,6,7,8,9,10,11,12]. Unlike other synthetic delivery vehicles, such as polymeric micelles and liposomes, VLPs are homogenous in their size distribution and are produced by inexpensive recombinant expression [1,2]. Further, VLPs are degradable in the body and have demonstrated few toxicity issues [1,5]. These protein-based nanomaterials also allow for site-selective modification through amino acid mutagenesis of natural or noncanonical amino acids into the protein backbone [6,13,14,15]. This site-selective conjugation allows for greater control over the location and amount of cargo loaded onto the VLPs, which can have significant effects on cancer targeting and delivery efficiencies.

Glioblastoma multiforme (GBM) is one of the deadliest and hardest to treat cancers, with over 17,000 new diagnoses per year [16]. While traditional small molecule chemotherapeutic approaches have largely failed to treat GBM, many nanomaterial-based approaches are being developed to enhance GBM treatment [16,17,18,19,20], although the reports of such approaches with virus-like particles is limited [21]. Alternative delivery routes are also being investigated for GBM treatment. Particularly, intratumoral injection of chemotherapeutics via convection-enhanced delivery has seen success in enhancing GBM treatment [22,23,24]. A constant pressure is maintained during the injection through a microfluidic pump that creates a fluid convection to facilitate a homogenous diffusion of the drug molecule throughout the targeted area [22]. After CED infusion, it is important for the chemotherapeutic agent to remain in the tumor tissue. We therefore hypothesized that VLP nanocarriers might be retained better in the tumor tissue after CED, as has been observed for polymeric constructs [22,25]. Additionally, the differing morphologies of VLPs could influence their tumor treatment efficacies in complex ways. This may be particularly important when developing drug delivery systems for glioblastoma treatment, as gliomas are known to form nanodimensional (50–200 nm) pores in their associated vasculature [26,27]. These pores may critically influence nanoparticle extravasation in a morphologically-dependent manner. Intriguingly, one report has noted differences in the pore sizes of malignant and benign glioma [27].

**Figure 1:**
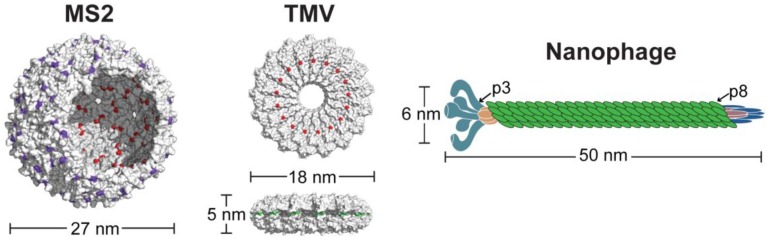
Panel of virus-like particles under evaluation. Three different nanocarriers composed of MS2 spheres, TMV disks and nanophage filamentous rods were tested to determine drug delivery efficacies. Each VLP contains reactive handles for bioconjugation, such as cysteines (**red**), reactive amines (**green**), or noncanonical p-aminophenylalanine moieties (**purple**).

To determine the efficacies of VLPs as nanocarriers for GBM treatment, a panel of distinct VLPs was evaluated for their drug delivery potential. This panel consisted of a 27 nm MS2 sphere, an 18 nm tobacco mosaic virus (TMV) disk and a 50 nm nanophage filamentous rod (Figure 1). MS2 VLPs have been widely used by our lab and others as drug delivery and diagnostic nanocarriers, as MS2 has a series of 2 nm pores along the surface that allow for interior capsid modification and subsequent drug release [2,6,13,28,29]. While MS2 has been extensively studied, it has not been tested for glioblastoma drug delivery. We recently reported a stable nanodisk composed of a double-arginine mutant of the tobacco mosaic virus [30]. These TMV disks maintained their structural assembly within all biologically-relevant conditions tested, which is uncommon for recombinantly expressed TMV mutants that lack their genomic material [31,32]. The TMV disks were further functionalized with chemotherapeutic cargo to showcase their potential as drug delivery vehicles, as disk-shaped nanomaterials have shown promise in other studies for their enhanced tumor accumulation and cell penetration [33,34]. The last member of the VLP panel was a nanoscale variation of the filamentous fd phage. The nanophage (NP) VLP was recently reported as a new potential nanocarrier [35] but has not previously been functionalized and evaluated for its drug delivery potential. This panel therefore encompasses disparate VLP morphologies, while maintaining relatively consistent dimensions of 15–50 nm. All three of the VLPs presented herein were functionalized with chemotherapeutic cargo to evaluate the efficacies of our VLP panel for drug delivery in glioblastoma models.

## 2. Materials and Methods

### 2.1. Reagents and Instruments

Unless otherwise noted, reagents were purchased from Sigma (St. Louis, MO, USA) and used without further purification. Sulfo-LC-SPDP was purchased from Thermo (Waltham, MA, USA). Water was deionized using the NANOpure purification system (Thermo). *N*-methylpyridinium-4-carboxaldehyde benzenesulfonate hydrate (Rapoport’s salt, RS) was obtained from Alfa Aesar (Ward Hill, MA, USA). NAP desalting columns were purchased from GE Healthcare (Marlborough, MA, USA). Spin concentrators with different molecular weight cutoffs (MWCO) were from Millipore (Billerica, MA, USA). Aminophenol-PEG_5k_-OMe [14], alkoxyamine-PEG_5k_-OMe [36] and DOX-EMCH [37] were synthesized as reported previously. EMEM media for cell culture was purchased from ATCC (Manassas, VA, USA). MTS Assay ((3-(4,5-dimethylthiazol-2-yl)-5-(3-carboxymethoxyphenyl)-2-(4-sulfophenyl)-2*H*-tetrazolium)) was purchased from Promega (Madison, WI, USA). Doxorubicin was purchased from Pfizer as a 2 mg/mL stock solution in saline. Liposomal Dox was purchased from SunPharma (Mumbai, India) as a 2 mg/mL solution.

Liquid chromatography mass spectrometry (LC/MS) analysis was performed using acetonitrile (Optima grade, 99.9%, Thermo Fisher), formic acid (99+%, Pierce, Rockford, IL, USA) and dd-H_2_O as mobile phase solvents. Electrospray ionization mass spectrometry (ESI-MS) of proteins was performed using an Agilent 1260 series liquid chromatograph outfitted with an Agilent 6224 time-of-flight (TOF) LC/MS system (Santa Clara, CA, USA). The LC was equipped with a Poroshell 300SB-C18 (5 μm particles, 1.0 mm × 75 mm, Agilent) analytical column. Data were collected and analyzed using Agilent MassHunter Qualitative Analysis B.05.00.

Sodium dodecyl sulfate-polyacrylamide gel electrophoresis (SDS-PAGE) was carried out on TRIS gels in a Mini-Protean apparatus from Bio-Rad (Hercules, CA, USA) or on Bis-TRIS gels in a Mini Gel Tank apparatus (Thermo), following the protocol from the manufacturer. The protein electrophoresis samples were heated for 10 min at 95 °C in the presence of β-mercaptoethanol to ensure reduction of any disulfide bonds. Gels were run for 35–60 min at 150–200 V in 2-(*N*-morpholino)ethanesulfonic acid (MES)—SDS buffer to allow good separation of the bands. Commercially available markers (Bio-Rad) were applied to at least one lane of each gel for assignment of apparent molecular masses. Visualization of protein bands was accomplished by staining with Coomassie Brilliant Blue R-250 (Bio-Rad). Quantification of the degree of modification was obtained by imaging on a Gel Doc™ EZ imager (Bio-Rad) and subsequent optical densitometry using the ImageJ (National Institutes of Health, Bethesda, MD, USA) or Image Lab (Bio-Rad) software.

UV-Vis spectrophotometer readings were carried out using a Cary 50 Bio Spectrophotometer (Agilent, Santa Clara, CA, USA) or a NanoDrop 1000 (Thermo Scientific). Analytical size exclusion was performed on an Agilent 1100 series HPLC equipped with a PolySep-GFC-P 5000 column (Phenomenex, Torrance, CA, USA), at a flow rate of 1 mL/min. Incucyte live cell imaging (Essen Bioscience, Ann Arbor, MI, USA) was used to monitor the cellular uptake of DOX-protein conjugates. An IVIS 50 Lumina imaging system (Perkin Elmer, Waltham, MA, USA) was used to measure in vivo and ex vivo bioluminescence.

### 2.2. Protein Purification and Expression

MS2 and TMV proteins were expressed and purified using previously published methods [15,28,30]. Nanophage was expressed and purified using an adapted method from a previously published report [35]. All proteins were purified using anion exchange chromatography with a diethylaminoethanol (DEAE) Sepharose column followed by size exclusion chromatography. Purity was confirmed by SDS-PAGE, LC/MS and HPLC-SEC. Detailed protocols of nanophage expression and purification are available in the Supporting Information.

### 2.3. Protein Modification

Protein modification reactions varied depending on the protein and the synthetic cargo. Full modification protocols of each protein and each modification are available in the Supporting Information. Typical modifications involved the use of 10 equiv maleimide, 40 equiv of isothiocyanate, 10–20 equiv of PEG and 1–2 equiv of DOX-EMCH. In the case of nanophage, the p8 monomer of the coat protein was the primary target for bioconjugation. All protein conjugates were purified via elution through a Nap desalting column followed by multiple rounds of spin concentration with a designated molecular weight cut-off (MWCO) that would allow for small-molecule flow-through but prevent protein flow-through. Protein conjugates were characterized by a combination of LC/MS, gel electrophoresis and HPLC-SEC (Supporting Figures S1–S4).

### 2.4. Cell Culture

U87-MG human glioblastoma cells were obtained from the UC Berkeley Cell Culture Facility. U87-MG human glioblastoma cells bearing the luciferase reporter (U87-Luc) were acquired from UCSF. Cells were cultured in DMEM containing phenol red (ATCC, Manassas, VA, USA) or DMEM without phenol red (Thermo, Waltham, MA, USA) with 10% fetal bovine serum (Omega Scientific, Tarzana, CA, USA) and 1% penicillin/streptomycin (Thermo) at 37 °C and 5% CO_2_.

### 2.5. Cell Viability Assays

U87-MG cells were trypsinized and diluted to a density of 50,000 cells/mL. An aliquot of 100 μL of cell stock was placed in each well of a 96 well plate (Corning, Corning, NY, USA) for a density of 5000 cells/well. The plate was incubated at 37 °C, 5% CO_2_ overnight. Following this, media was removed from the plate and 100 μL of appropriate sample stocks media was added. The cells were incubated at 37 °C, 5% CO_2_ for 3 d. The media containing the sample was removed from the well and 100 μL of MTS media (20% MTS in media) was added to each well and incubated for 1–3 h. An Infinite 200 Pro plate reader (Tecan, Switzerland) was used to measure the MTS absorbance at 490 nm. Cell viability was calculated as an absorbance percent relative to the untreated cell control. The experiment was performed in triplicate. 

### 2.6. Cell Uptake Studies

Cell uptake studies were performed as previously described [30]. U87-MG cells were trypsinized and diluted to a density of 50,000 cells/mL. An aliquot of 200 μL of cell stock was placed in a well of a 96 well plate (Corning) for a density of 10,000 cells/well. The plate was incubated at 37 °C, 5% CO_2_ for 48 h. Following this, media was removed from the plate and 200 μL of appropriate sample stocks (standardized to 1 μM DOX) in phenol free media was added. The cells were incubated at 37 °C, 5% CO_2_ for 48 h. Incucyte Zoom Live-Cell Analysis System (EssenBio, Ann Arbor, MI, USA) was used to collect images every hour post incubation. Phase and green fluorescence images at 20× magnification were collected, capitalizing on the intrinsic fluorescence of doxorubicin. The images were processed using Incucyte Zoom proprietary software v.2016A and the Top-Hat background subtraction algorithm (radius 10 µm, threshold 0.5% of green calibration dye signal, GCU) was used to define the boundaries of the cells (green objects). The mean fluorescence intensities of 4 images taken in each well were plotted against the incubation time.

### 2.7. Tumor Growth and Survival Studies in Glioblastoma Models

All animal procedures were performed according to a protocol approved by the University of California San Francisco Institutional Animal Care and Use Committee (IACUC). Five-week old athymic (nude) female mice weighing 18–23 g were purchased from Simonsen Labs (Gilroy, CA, USA). For tumor inoculation, 3 × 10^5^ U87-Luc glioblastoma cells with luciferase reporter gene were implanted intracranially [38].

Mice bearing U87-Luc intracranial tumors in sets of 9 animals per study group were injected via convection enhanced delivery (CED) with 10 μL of PBS, DOX, Lipo-Dox, or VLP-DOX conjugates in sterile saline, at day 11 post-implantation. CED infusion cannula were made with silica tubing (Polymicro Technologies, Phoenix, AZ, USA) fused to a 0.1 mL syringe (Plastic One, Roanoke, VA, USA) with a 0.5 mm stepped-tip needle that protruded from the silica guide base. Syringes were loaded with the agents and attached to a microinfusion pump (Bioanalytical Systems, Lafayette, IN, USA). The silica cannula attached with a infusion pump was lowered to a 4 mm depth through a skull hole, which was made by skull puncture with a coordination of 3 mm to the right from bregma and just on top of the coronal suture (the same region in the caudate putamen at which tumor cells were injected). The agents were infused at a rate of 1 μL/min until a volume of 10 μL had been delivered. Cannulae were removed 5 min post completion of infusion.

Tumor size was monitored using the luciferase reporter system and measuring bioluminescence on an IVIS 50 Lumina system (Supporting Figure S5). Mice were euthanized when tumor burden reached levels determined by IACUC guidelines. Kaplan Meier survival curves were plotted based on these survival points. Log rank tests were performed on the Kaplan Meier curves to gauge statistical significance. When dividing mice into small and large tumor cohorts, a Bioluminescence Intensity (BLI) of 10^7^ was taken as the cutoff, as this was the standard midpoint across groups when assessing tumor size prior to CED injection. Statistical comparison between small and large tumor cohorts was performed using log rank tests of the Kaplan Meier survival curves for each cohort. A full listing of tumor size in all mice that were sacrificed due to tumor burden is available in Supporting Figure S6. Detailed side-by-side survival curves of key cohorts are available in Supporting Figure S7.

## 3. Results and Discussion

### 3.1. Chemical Modification of VLPs for Drug Delivery

In order to investigate the potential of our VLP panel as nanocarriers for glioblastoma treatment, we synthesized a series of conjugates with the chemotherapeutic molecule doxorubicin (DOX). Doxorubicin is an anthracycline antibiotic commonly used for the treatment of many types of cancer. However, its systemic toxicity (especially cardiac toxicity) and poor penetration through the blood-brain barrier limit its use in the treatment of brain tumors [16,39]. Several different formulations of doxorubicin have been developed, including PEGylated liposomal doxorubicin (Doxil) [40]. Unfortunately, none of these agents showed activity in clinical trials investigating its use to treat brain cancer [16]. We hypothesized that VLP carriers could enhance the delivery of DOX for glioblastoma treatment. By attaching doxorubicin to the protein scaffolds through acid-labile linkers, the release and distribution of DOX can be controlled to achieve more effective drug release and tumor treatment.

**Figure 2:**
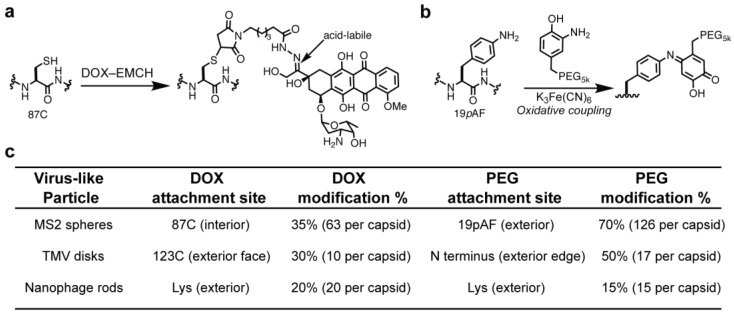
Doxorubicin (DOX) and PEG conjugation to virus-like particles for drug delivery studies. (**a**) Bioconjugation scheme for the modification of MS2 with (**a**) DOX–EMCH and (**b**) PEG. DOX–VLP conjugates contain an acid-labile hydrazone linkage, which is anticipated to cleave upon VLP endocytosis and degradation. Modification schemes for TMV and nanophage are available in the Supporting Information. (**c**) Each VLP was modified with DOX–EMCH using maleimide addition to cysteine residues (MS2, TMV) or to thiols synthetically installed onto lysine residues (nanophage) as shown in Figure S1. All VLPs were modified with PEG using either oxidative couplings (MS2, TMV) or NHS ester reactions (nanophage) to improve biodistribution.

The ketone moiety on DOX was modified via a condensation reaction with *N*-ε-maleimidocaproic acid hydrazide (EMCH), as previously described (Figure S1) [30,37]. The maleimide moiety of DOX–EMCH was reacted with thiol residues on MS2, TMV and Nanophage VLPs to produce VLP–DOX conjugates (Figure 2). This modification was shown to retain the cytotoxic activity of doxorubicin upon hydrolysis of the hydrazone linkage between EMCH and DOX, which releases DOX in its native state. It is anticipated that the DOX will release upon endocytosis of the VLP–DOX conjugates, as the late endosome and subsequent lysosome have internal pH in the range of pH 4.5–5.5, which is sufficient to cleave the hydrazone linkage. For the nanophage, native Lys residues were first converted to thiols using Traut’s reagent [41]. The VLPs were further modified with PEG_5k_, as this is a standard modification for improving biodistribution and minimizing the immune response against VLPs [3,4,28]. Additionally, VLP PEGylation helped improve the solubility of the protein constructs, as DOX is a relatively hydrophobic molecule.

### 3.2. Evaluation of VLP–DOX Conjugates In Vitro

**Figure 3:**
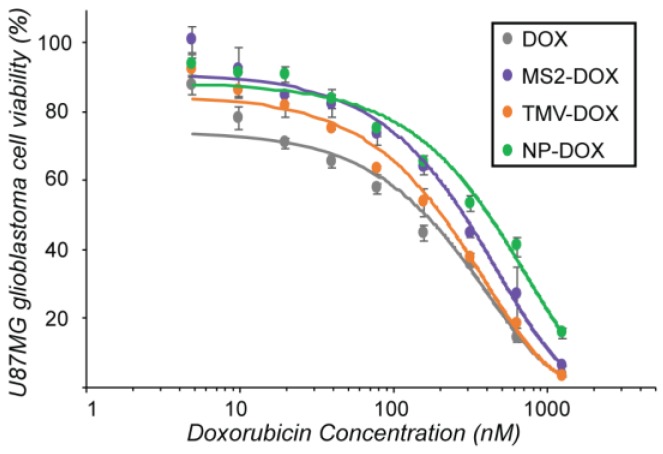
VLP delivery of doxorubicin to U87-MG glioblastoma cells. U87-MG glioblastoma cells were treated with varying amounts of VLP–DOX conjugates for 72 h. Significant cell death was observed for all VLPs tested. Treatment with the VLPs alone displayed no toxicity (data not shown).

After chemical modification, the VLP–DOX conjugates were evaluated for their effects on cell viability. U87-MG glioblastoma cells were incubated with the agents at different concentrations for 72 h to allow sufficient time for DOX release from the VLP conjugates upon endocytosis and a cell viability assay was performed (Figure 3). All nanocarriers showed a similar dose-response curve and had comparable efficiency compared to the free drug, suggesting that the drug was released and was able to reach the nucleus and induce cell death.

**Figure 4:**
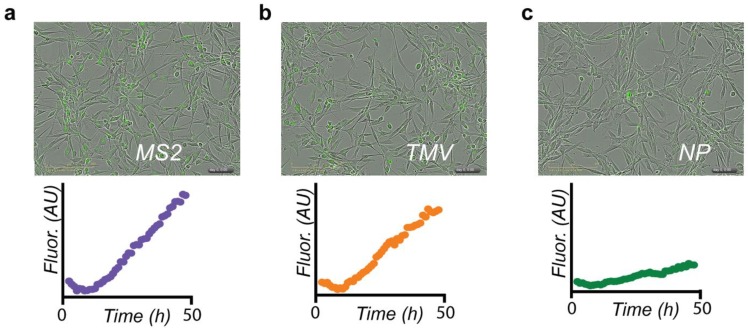
VLP-DOX uptake into U87-MG cells at 48 h. Uptake kinetics of VLP-DOX conjugates into U87-MG glioblastoma cells were monitored in the green channel to detect DOX fluorescence. Data are presented with consistent scaling. Initial intensities are artificially high due to the presence of autofluorescent compounds in the cell media. Upon photobleaching, the FLI drops to more accurate baselines. Both (**a**) MS2 and (**b**) TMV demonstrated significant cellular uptake, while the uptake of (**c**) nanophage (NP) was markedly slower. The TMV data appeared previously in reference [30].

In order to understand the uptake kinetics of DOX conjugates in glioblastoma cells, cell uptake was monitored using live cell imaging. Cells were incubated with VLPs bearing a standard dose of 1 µM DOX and images were collected every hour for 48 h to assess the rate of VLP–DOX accumulation. Both MS2–DOX and TMV–DOX displayed fast uptake into cells, while the nanophage–DOX conjugates displayed slower uptake in comparison (Figure 4). This may be due to different cellular uptake efficiencies between nanoparticle morphologies, as has been observed previously [33,42,43,44,45]. Overall, despite subtle differences in uptake kinetics and drug delivery efficacies, all three VLPs were amenable to DOX modification and were able to deliver their chemotherapeutic payload in cell culture effectively.

### 3.3. Convection-Enhanced Delivery of VLP–DOX Conjugates

**Figure 5:**
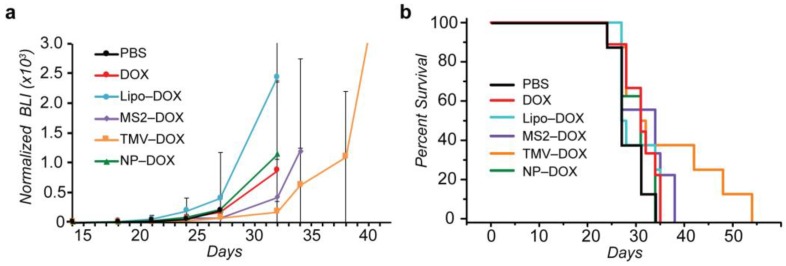
Evaluation of VLP panel for glioblastoma treatment. MS2 spheres, TMV disks and nanophage rods bearing DOX payloads of 20 µg/kg were injected via convection-enhanced delivery infusion (CED) into U87-MG glioma-bearing mice. (**a**) Tumor growth analysis suggested modest tumor growth inhibition from MS2 and TMV-treated groups. (**b**) Kaplan-Meier survival curves of mice with U87-MG glioma. The survival curve results suggest improved efficacy with TMV–DOX treatment.

Each of the three VLP–DOX conjugates was evaluated for its ability to treat glioblastoma after CED injection. VLP-DOX conjugates were compared to PBS, DOX and liposomal DOX (Lipo–DOX) treatment via CED injection 11 days after intracranial tumor implantation (Figure 5). While the payload of DOX per nanocarrier was variable, each glioma-bearing mouse was injected with a standardized DOX payload of 20 µg/kg and tumor size was monitored via bioluminescence intensity (BLI, Figure 5a). The tumor growth of MS2 and TMV-treated mice demonstrated some growth inhibition, although the large variability in tumor reduction within groups led to no statistical difference between treatment groups. Kaplan Meier survival analysis of treated mice suggested improved outcomes for mice treated with TMV–DOX conjugates, as three out of eight mice survived past 40 days, whereas for the PBS-treated group, no mouse survived past 34 days (Figure 5b). This is particularly encouraging, as the CED injected dose of DOX was orders of magnitude lower than the milligrams per kilogram of doxorubicin typically injected intravenously [18,46]. However, log rank analysis of survival curves of TMV and PBS-treated mice did not demonstrate statistical significance between the groups for the full set of animals (*p* = 0.052).

**Figure 6:**
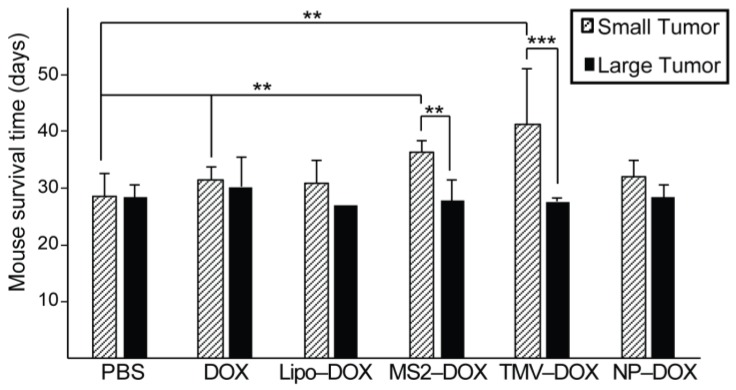
Tumor size dependence of mouse response to VLP treatment. Treatment groups could be divided into two categories based on tumor size as measured by bioluminescence intensity (BLI). Small tumors (as defined by a BLI < 107 AU) showed significantly increased response to CED treatment in the case of TMV and MS2 treated mice when compared to larger tumors (BLI > 107 AU). This BLI cutoff led to a relatively even distribution of mice within each treatment group with the exception of Lipo–DOX, where only two mice had large tumors. Both MS2–DOX and TMV–DOX treatment of small tumors led to significantly enhanced survival when compared to PBS treatment. ** *p* < 0.05. *** *p* < 0.01 as measured by log rank test of survival points. See Supporting Information Figures S5–S7 for the tumor size and survival data for individual animals.

The glioma-bearing mice under study could be divided into two cohorts: one with small tumors prior to CED injection and one with larger tumors. The cutoff between large and small tumor size was determined by analyzing the bioluminescence intensities (BLI) of treatment groups and arriving at a general midpoint of 10^7^ BLI (Supporting Figure S6). This cutoff allowed for relatively even distributions between large and small tumors within each treatment group, with the exception of Lipo–DOX, which only had two large tumors within that group. Analysis of survival times between large and small tumors within treatment groups revealed significant differences for MS2–DOX and TMV–DOX treated groups (Supporting Figure S7), with smaller tumors responding better to the VLP treatment (Figure 6). This analysis reveals that the size of the glioblastoma could significantly influence the drug delivery efficacy of VLPs. This may be due to differences in VLP diffusion within large versus small tumors, or due to vasculature differences between large and small tumors that could influence drug delivery efficacies [47,48,49]. It is also possible that larger tumors would require higher injected doses to reduce the tumor burden and increase survival times. Further analysis of these tumor size cohorts revealed that both MS2–DOX and TMV–DOX treatments significantly increased the survival times of small tumor-bearing mice, when compared to PBS controls, with TMV–DOX treatment displaying the highest improved survival (Figure 6). Lastly, mice treated with MS2–DOX displayed significantly improved survival rates when compared to DOX treatment alone. Taken together, these findings underscore the potential of VLPs as nanocarriers for GBM treatment via CED, especially for the treatment of smaller tumors and illustrate some of the morphological effects that are at play in VLP drug delivery.

## 4. Conclusions

We present here an analysis of three morphologically distinct virus-like particles on their ability to deliver drugs to glioblastoma. A panel of virus-like particles representing spheres, disks and filamentous rods was modified with chemotherapeutic cargo and PEGylated via orthogonal bioconjugation strategies. These VLPs were taken up into cancer cells and effectively released their chemotherapeutic payload in cell culture. The VLPs were further tested in glioma-bearing mouse models and showed significant differences in their efficacies for glioblastoma treatment via CED injection. Importantly, glioblastoma-bearing mice treated with TMV-DOX demonstrated improved survival. This is especially encouraging given the low dose of DOX delivered to the mice. Lastly, tumor size dependence of mouse survival was observed for VLP-treated mice, indicating that the tumor environment could have significant implications for GBM treatment via protein-based nanomaterials. Future studies will focus on the evaluation of these VLPs in other tumor models to determine if different tumor types require different VLPs for effective delivery of a chemotherapeutic payload. These studies will help expand our understanding of the effects of virus-like particle morphology on drug delivery, which may have significant implications in the growing field of nanomedicine and help lead to the development of functional protein-based nanomaterials for cancer treatment.

## Supplementary Materials

The following are available online at http://www.mdpi.com/2079-4991/8/12/1007/s1, Figure S1: Modification Scheme for TMV and Nanophage Conjugation, Figure S2: LC/MS characterization of DOX-modified VLPs, Figure S3: HPLC-SEC of Nanophage-PEG_5k_-DOX, Figure S4: Protein gel characterization of VLP-PEG_5k_-DOX conjugates, Figure S5: Individual tumor growth plots for each treatment group, Figure S6: Tumor size distribution of glioblastoma-bearing mice and survival points, Figure S7: Kaplan Meier Survival Curves of small and large tumor cohorts.
