# Supplementary material for: Evaluation of Three Morphologically Distinct Virus-Like Particles as Nanocarriers for Convection-Enhanced Drug Delivery to Glioblastoma
Source: Nanomaterials (Basel). 2018 Dec 5;8(12):1007. doi: 10.3390/nano8121007 (PMC6315496; doi:10.3390/nano8121007)
Supplement: Supplementary file 1 [file nanomaterials-08-01007-s001.pdf]

Evaluation of three morphologically distinct virus-like particles as nanocarriers for convection-enhanced drug delivery to glioblastoma

Joel A. Finbloom,[1,†] Ioana L. Aanei,[1,†] Jenna M. Bernard,[1,†] Sarah H. Klass,[1] Susanna K. Elledge,[1] Kenneth Han,[1] Tomoko Ozawa,[3] Theodore P. Nicolaides,[4] Mitchel S. Berger,[3] and Matthew B. Francis.*,[1,2]

[1]Department of Chemistry, University of California, Berkeley, and [2]Materials Sciences Division, Lawrence Berkeley National Laboratories, Berkeley, California 94720. [3]Department of Neurological Surgery, University of California, San Francisco, San Francisco, California, 94158. [4]Department of Pediatrics, NYU Langone Medical Center. New York, New York, 10016.

[†]These authors contributed equally

**mbfrancis@berkeley.edu*

SUPPORTING INFORMATION

Table of Contents

S1. Expression and Purification of Nanophage S2

S2. Detailed Protein Modification Protocols S2

S3. Supplemental Figures S3

Figure S1. Modification Scheme for TMV and Nanophage Conjugation S3

Figure S2. LC/MS characterization of DOX-modified VLPs. S4

Figure S3. HPLC-SEC of Nanophage-PEG_{5k}-DOX. S4

Figure S4. Protein gel characterization of VLP-PEG_{5k}-DOX conjugates. S5

Figure S5. Individual tumor growth plots for each treatment group. S5

Figure S6. Tumor size distribution of glioblastoma-bearing mice and survival points. S6

Figure S7. Kaplan Meier Survival Curves of small and large tumor cohorts. S7

S1. Expression and purification of nanophage

The nanophage procedure was adapted from Sattar *et al*. The nanophage used in this study is unable to replicate on its own during expression. To overcome this limitation, a "helper" phage is first expressed and purified to infect *E.coli* cells and facilitate the expression of the non-replicative nanophage. The "helper" phage is then removed from the mixture, leaving only nanophage.

Expression of the "helper" phage needed to produce nanophage proteins. TG1 cells (with no plasmids) were inoculated in 10 mL 2xYT media and incubated at 37 °C overnight at 250 rpm. TG1 cells transformed with R779 plasmid were inoculated in 5 mL 2xYT media and incubated at 37 °C overnight. The TG1 cells were diluted to 1 L 2xYT and allowed to grow to OD600 = 0.1 (1-3 h). A 1 mL R779 portion of a cell culture expressing phage was added to 2xYT media containing the TG1 cells to be infected and incubation was continued for an additional 12-16 h at 37 °C, 250 rpm. The cells were centrifuged at 12,000x *g* at 4 °C for 20 min. The pellet was discarded and the supernatant was mixed with solid PEG_{8k} and NaCl to achieve a final concentration of 2.5% w/v PEG and 0.5 M NaCl (25 g PEG and 29.2 g NaCl per L of supernanant). The phage was precipitated on ice overnight, then spun at 12,000x *g* at 4 °C for 20 min. The pellet was resuspended in 10-25 mL PBS and centrifuged at 10,000x *g* for 10 min to remove any residual bacterial cells. The supernatant was mixed with PEG_{8k} and NaCl to achieve 4% w/v PEG_{8k} and 0.5 M NaCl and was precipitated on ice for at least 2 h. After centrifugation at 16,000x *g*, 4 °C for 45 min, the pellet was resuspended in PBS and stored at 4 °C.

Expression of nanophage. K1030 *E.coli* were transformed with a PNJB07 plasmid and were inoculated in 5 mL 2xYT with 100 µg/mL ampicillin. The cells were allowed to grow at 37 °C, 280 rpm overnight. Several dilutions (10^{-4}-10^{-8}) were plated on LB plates to ensure separated colonies upon overnight incubation at 37 °C. The colonies were inoculated in 10 mL 2xYT with 100 µg/mL ampicillin and allowed to grow at 37 °C, 280 rpm overnight. The cells were then transferred to 1 L 2xYT containing 100 µg/mL ampicillin and grown at 200 rpm until OD600 = 0.2 (2-2.5 h). The shaking of the incubator was stopped, and the incubation was continued until the OD600 reached 0.3. At this point, the cells were infected with "helper" phage (from the cells prepared as described above) with multiplicity of infection (m.o.i.) of 50-100 phage/cell and incubation was continued for 15 min without shaking, then for 4 h at 37 °C, 200 rpm. After the OD600 reached 1.8, the cells were centrifuged at 12,000x *g*, 4 °C for 20 min. The supernatant was transferred into a new container and the helper phage was precipitated using a final concentration of 2.5% w/v PEG_{8k} and 0.5 M NaCl on ice overnight. The helper phage was removed by centrifugation at 16,000x *g*, 4 °C for 45 min. The supernatant was mixed with PEG to achieve 15% w/v PEG_{8k} final concentration and precipitated on ice overnight. The nanophage was removed by centrifugation at 16,000x *g*, 4 °C for 60 min, then resuspended in 10-25 mL buffer.

S2. Detailed Protein Modification Protocols

Synthesis of MS2-PEG_{5k}-DOX. A solution of 140 µM MS2 T19*pa*F N87C in 10 mM phosphate buffer pH 7.2 was mixed with DOX-EMCH from a stock in DMSO (0.75 equiv). The reaction was incubated for 3 h at room temperature. The excess DOX was removed by using Nap-25 desalting columns and 100 kDa MWCO spin concentrators. For the synthesis of the MS2–DOX conjugate, $K_3[Fe(CN)_6]$ was used to prevent the oxidation of the DOX molecule in the presence of $NaIO_4$. The final concentrations were 100 µM for the MS2 monomer, 500 µM aminophenol-PEG_{5k}-OMe and 5 mM of $K_3[Fe(CN)_6]$. The reaction was allowed to proceed for 1 h at room temperature. The reaction mixture was loaded onto a Nap-5 desalting column and eluted with phosphate buffer pH 7.2. Subsequent concentration and additional removal of the excess reagents were achieved using 100 kDa MWCO spin concentrators. The conjugate was stored frozen in the presence of 500 equiv of trehalose at -20 °C until use to prevent DOX hydrolysis. Right before use, the stock was thawed and the trehalose was removed using 100 kDa MWCO spin concentrators.

Synthesis of TMV-PEG$_{5k}$-DOX. To a solution of TMV (RR mutant, PAGSYS N-terminus, S123C, T104K, 100 µM) in 20 mM pH 7.5 sodium phosphate buffer, 1 equiv of DOX-EMCH (2 µL of 100 mM stock solution) was added. The solution was incubated for 1 h at room temperature, at which point an aliquot was taken for LC-MS analysis. The solution was then spin concentrated 3-5 times into 25 mM pH 6.5 sodium phosphate buffer using a 30 kDa MWCO spin concentrator. Aminophenol-PEG$_{5k}$-OMe (5 equiv) and $K_3[Fe(CN)_6]$ (50 equiv) were then added and the reaction mixture was incubated for 30 min at room temperature. The excess reagents were removed using 3-5 rounds of spin concentration with 30 kDa MWCO spin concentrators. The conjugate was stored frozen at -20 °C until use.

Synthesis of nanophage-PEG$_{5k}$-DOX. To a solution of nanophage in 100 mM phosphate buffer pH 8 (25 µM final concentration p8), 25 equiv of 2-iminothiolane were added. The reaction mixture was incubated at room temperature for 15 min. Excess reagent was removed using 100 kDa spin concentrators. After the formation of the thiol groups, 5 equiv of maleimide-PEG$_{5k}$-OMe (JenKem, Plano, TX) were added and allowed to react at room temperature for 45 min. Excess reagent was removed using 100 kDa spin concentrators. DOX-EMCH was then added (2 equiv) and incubated with the reaction mixture for 45 min at room temperature. The excess reagents were removed using 3-5 rounds of spin concentration with 100 kDa MWCO spin concentrators. The conjugate was stored frozen at -20 °C until use.

S3. Supplemental Figures

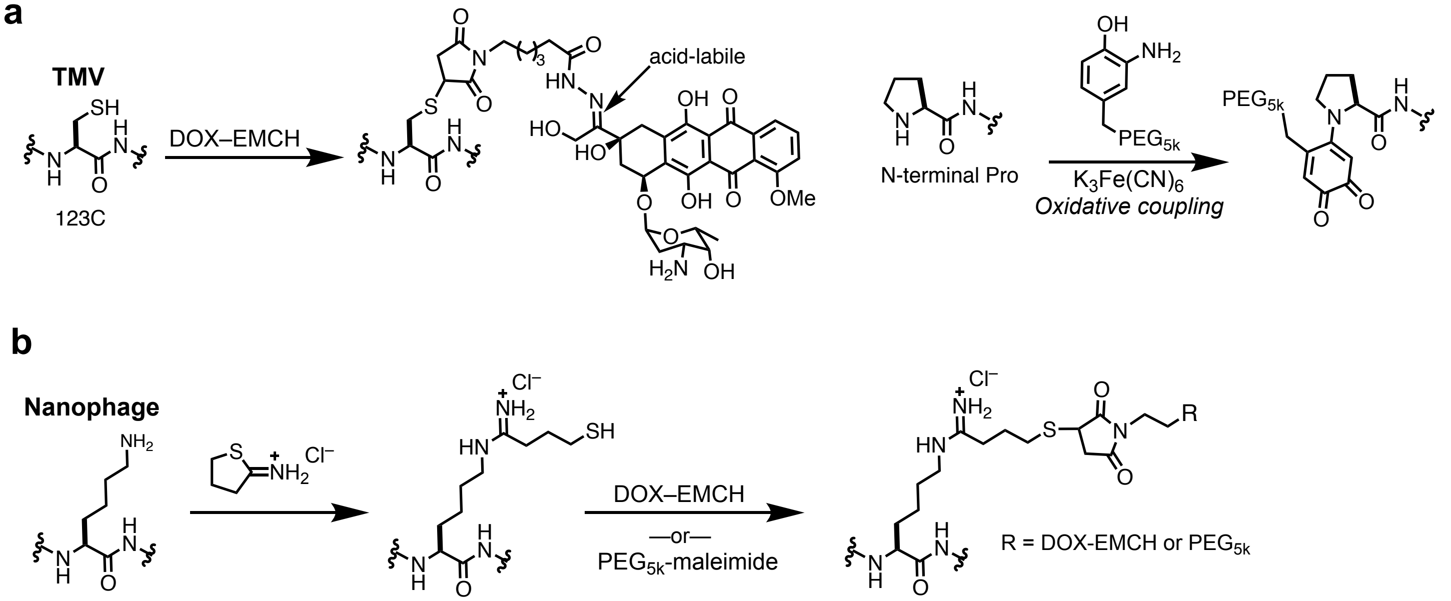

Figure S1. Modification scheme for TMV and Nanophage conjugation with DOX and PEG$_{5k}$. (a) TMV was modified at the 123C position with DOX-EMCH, followed by oxidative coupling with PEG$_{5k}$-aminophenol. (b) Nanophage surface lysines were converted to reactive thiols using Traut's reagent, followed by modification with either DOX-EMCH or PEG$_{5k}$-maleimide

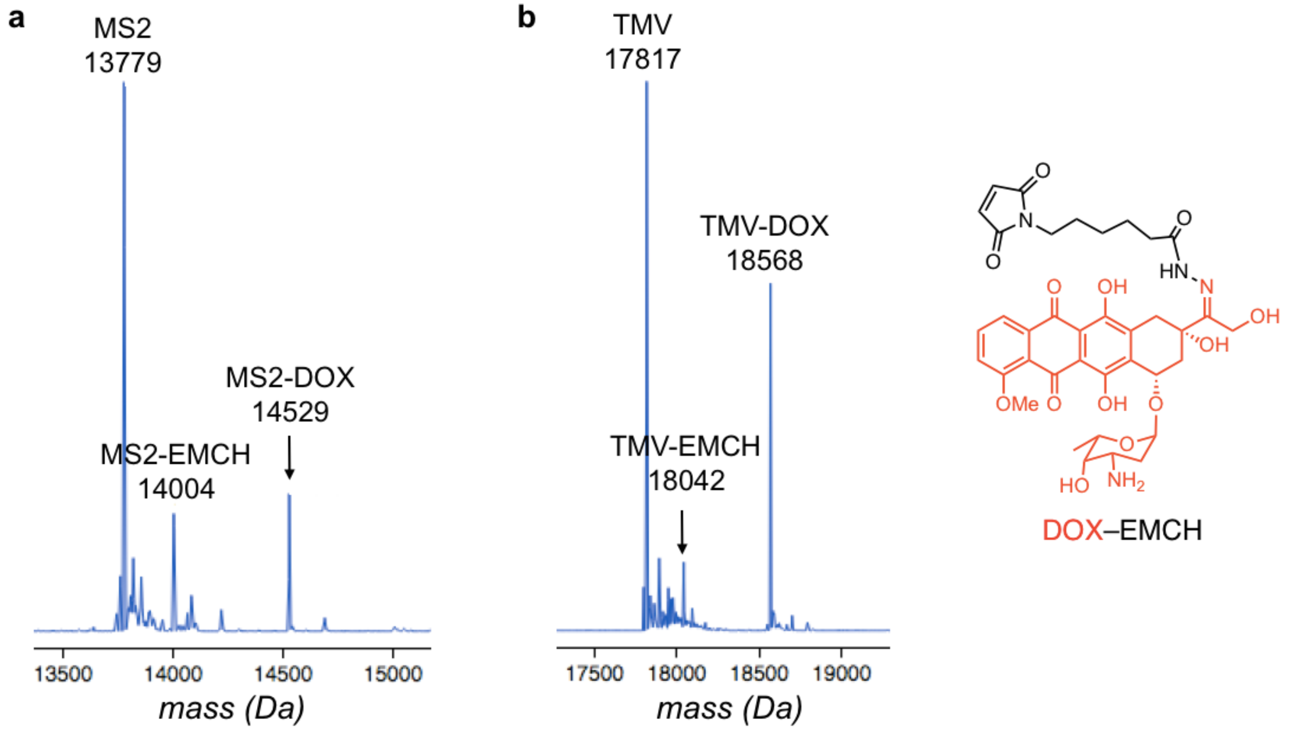

Figure S2. LC/MS characterization of DOX-modified VLPs. Mass spectra of (a) MS2 and (b) TMV modified with DOX-EMCH maleimide. Both VLPs demonstrated efficient levels of modification, as well as some DOX hydrolysis to the VLP-EMCH product that likely occurred due to the acidic mobile phase of the LC/MS. Nanophage-DOX conjugates were analyzed by HPLC-SEC (Figure S5) because the PEGylation and DOX modification occurred in one pot and PEGylated VLPs are unable to be analyzed via LC/MS.

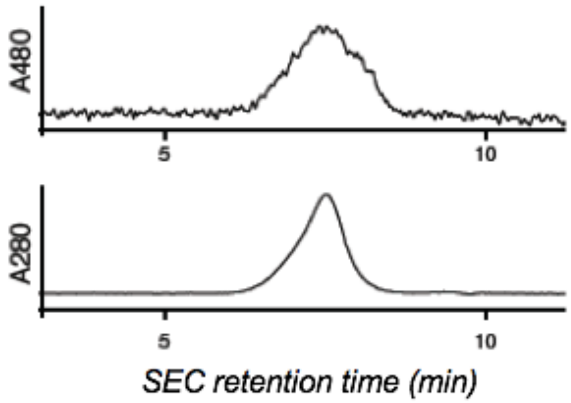

Figure S3. HPLC-SEC of Nanophage-PEG$_{5k}$-DOX. Nanophage-DOX conjugation was confirmed using HPLC-SEC to monitor the 480 nm absorbance of DOX, which co-eluted with the nanophage monitored at A280 nm.

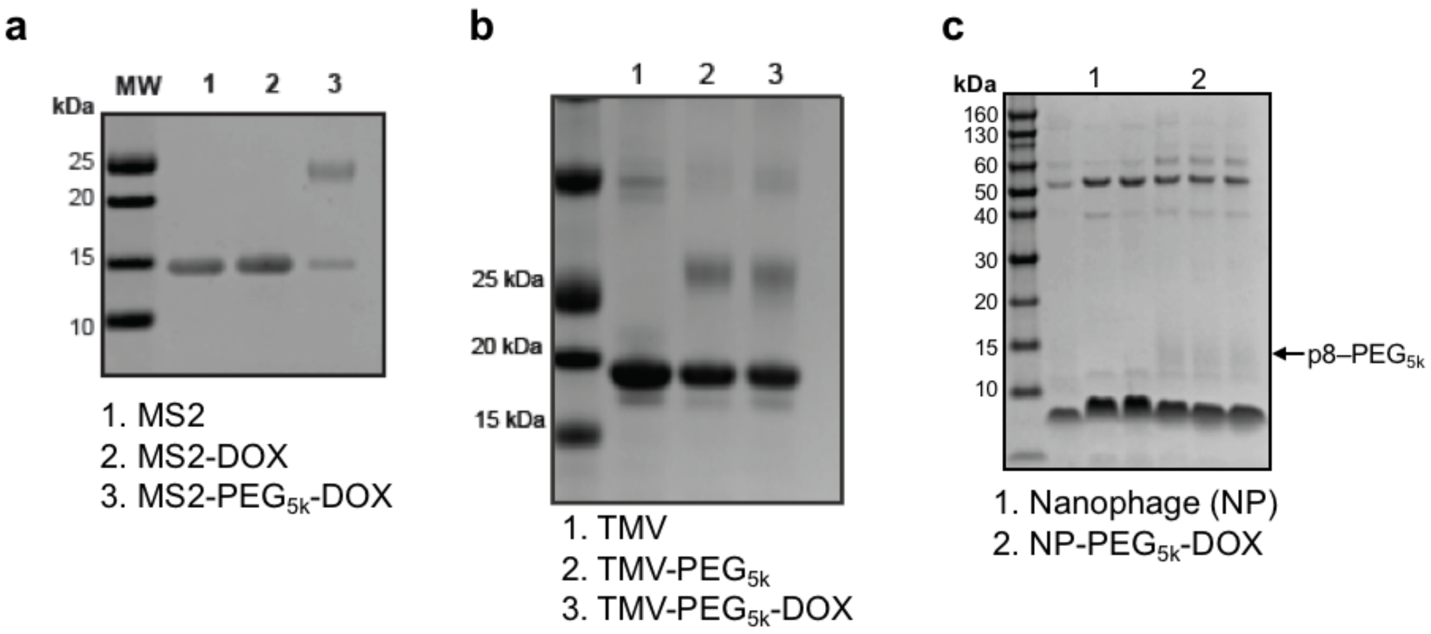

Figure S4. Protein gel characterization of VLP-PEG$_{5k}$-DOX conjugates. PEGylation of (a) MS2, (b) TMV, and (c) Nanophage (NP) p8 PEGylation was confirmed by gel electrophoresis and percent modifications were determined in imageJ using gel densitometry.

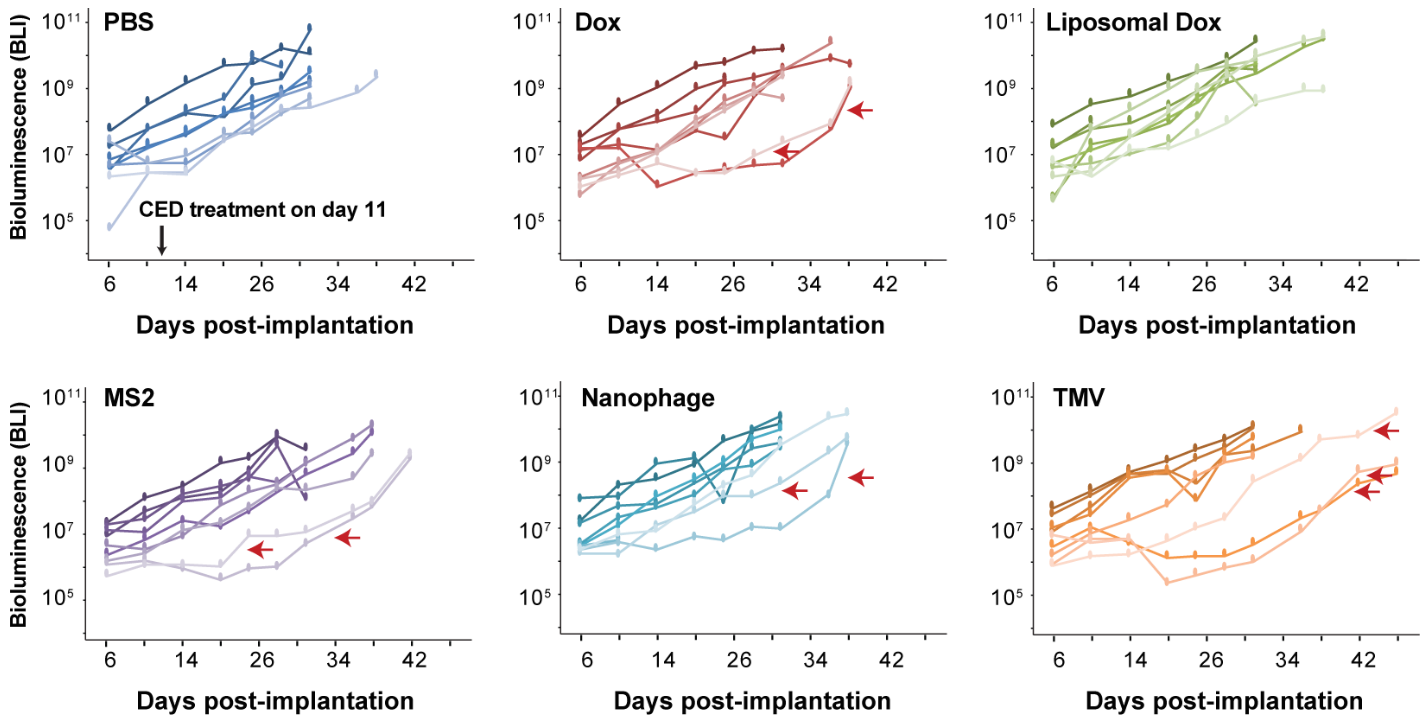

Figure S5. Individual tumor growth plots for each treatment group. Arrows indicate mice that displayed tumor growth suppression.

PBS Treatment

Mouse ID	Tumor BLI	Survival Day
696	8.09E+04	34
655	4.58E+06	24
685	4.95E+06	27
704	5.44E+06	31
664	8.42E+06	27
673	1.95E+07	27
643	2.75E+07	31
665	5.89E+07	27

DOX Treatment

Mouse ID	Tumor BLI	Survival Day
676	7.91E+05	31
708	1.31E+06	35
674	2.17E+06	31
705	2.51E+06	32
688	8.18E+06	28
666	1.45E+07	34
671	1.73E+07	35
650	2.36E+07	24
702	3.84E+07	28

Lipo-DOX Treatment

Mouse ID	Tumor BLI	Survival Day
697	5.07E+05	27
646	6.20E+05	35
680	2.58E+06	34
648	4.78E+06	27
698	6.51E+06	28
684	6.99E+06	35
641	2.07E+07	27
681	7.88E+07	27

MS2 Treatment

Mouse ID	Tumor BLI	Survival Day
701	1.33E+06	38
649	2.83E+06	38
658	3.67E+06	35
706	5.47E+06	34
645	1.01E+07	34
672	1.94E+07	24
642	2.84E+07	27
669	4.06E+07	27
670	4.50E+07	27

TMV Treatment

Mouse ID	Tumor BLI	Survival Day
695	9.98E+05	42
709	1.04E+06	54
663	2.10E+06	31
677	3.64E+06	48
707	9.45E+06	32
662	1.30E+07	28
651	3.05E+07	27
657	4.83E+07	27

Nanophage Treatment

Mouse ID	Tumor BLI	Survival Day
689	2.26E+06	34
660	2.89E+06	34
659	2.93E+06	34
687	3.67E+06	31
690	4.39E+06	27
693	1.66E+07	31
653	1.99E+07	27
647	9.12E+07	27

Figure S6. Tumor size distribution of glioblastoma-bearing mice and survival points. Treatment groups could be divided into two cohorts depending on tumor size (small versus large with a BLI cutoff of 10^7 as a relative midpoint within all groups). The BLI data presented in the tables were measured at 6 d post tumor implantation.

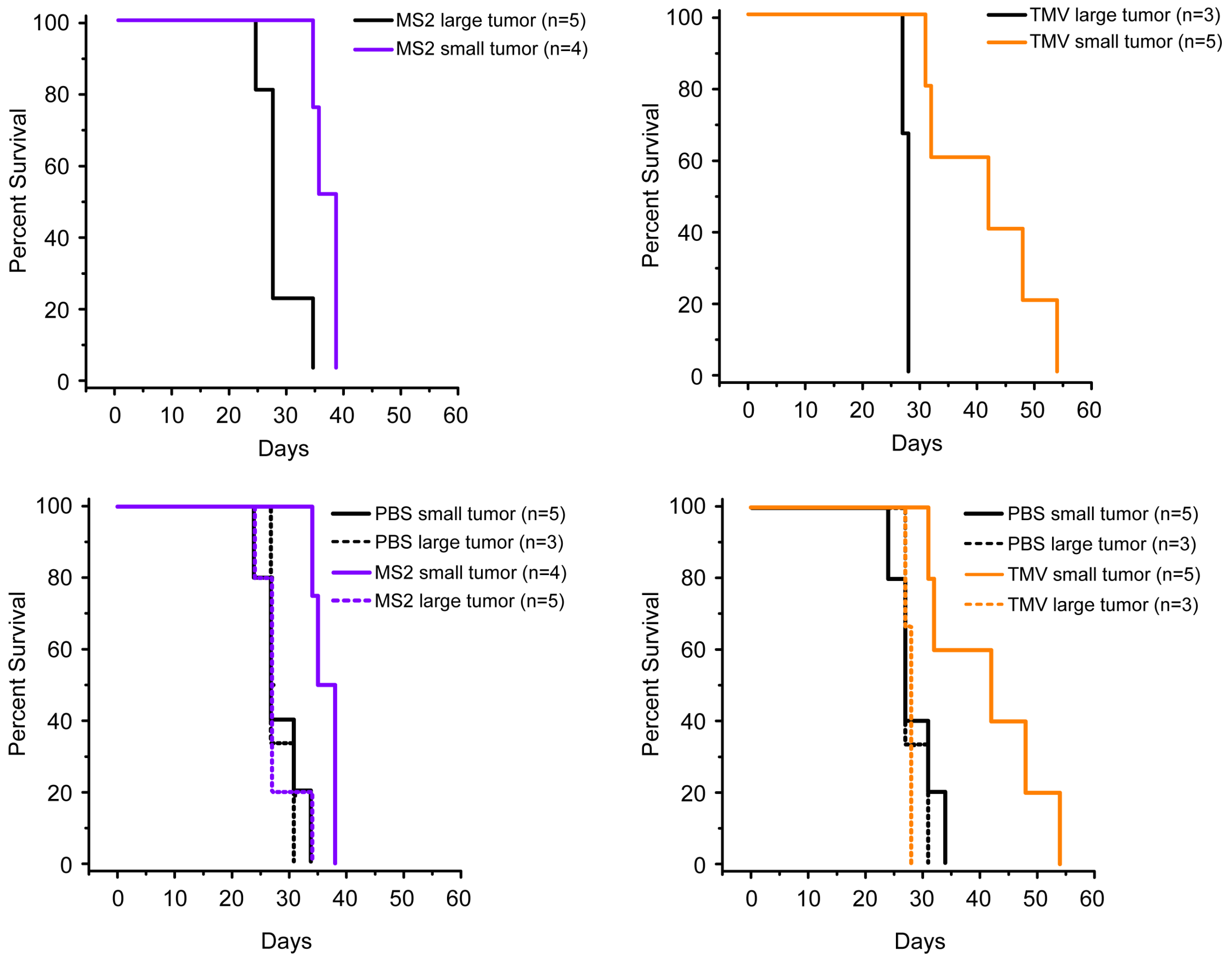

Figure S7. Kaplan Meier Survival Curves of small and large tumor cohorts. Log rank tests between cohorts were performed. Statistically significant differences, as determined by long rank analysis, were observed between the survival curves of large and small tumors for TMV ($p = 0.0067$) and MS2 ($p = 0.012$) treated mice. Statistically significant differences were also observed between PBS-treated small tumors and small tumors treated with MS2 ($p = 0.011$) or TMV ($p = 0.027$).
